# The interrelation of endothelial function and microvascular reactivity in different vascular beds, and risk assessment in hypertension: results from the Doxazosin–ramipril study

**DOI:** 10.1007/s00380-018-1265-7

**Published:** 2018-09-22

**Authors:** Andreas Jekell, Majid Kalani, Thomas Kahan

**Affiliations:** 1 Division of Cardiovascular Medicine, Department of Clinical Sciences, Danderyd Hospital, Karolinska Institutet, 182 88 Stockholm, Sweden; 2 Department of Cardiology, Danderyd University Hospital Corp, Stockholm, Sweden

**Keywords:** Cardiac hypertrophy, Endothelial function, Hypertension, Prognosis, SCORE, Skin microcirculation

## Abstract

There are several non-invasive methods to study endothelial function, but their interrelation and association to cardiovascular risk have not been well evaluated. We studied macrovascular and microvascular endothelial function simultaneously in different vascular beds in relation to cardiovascular mortality risk (Systematic Coronary Risk Evaluation, SCORE) and hypertension induced cardiac organ damage, and their interrelationship. The study investigated 71 hypertensive patients by forearm post-ischemic flow-mediated vasodilation, pulse wave analysis (applanation tonometry) and beta 2-adrenoceptor agonist stimulation for changes in reflection index, skin microvascular reactivity by laser Doppler fluxmetry with iontophoresis and heat-induced hyperaemia, and coronary microvascular function by subendocardial viability ratio (derived from pulse wave analysis). Flow mediated vasodilation related inversely to SCORE (*r* =  0.34, *P* = 0.011). Adding microalbuminuria and pulse wave velocity strengthened the associations. Pulse wave reflection changes did not relate to SCORE. Skin microvascular reactivity related inversely to SCORE (peak flux change to sodium nitroprusside *r* =  0.29, *P* = 0.033, and to heating *r* =  0.31, *P* = 0.018). Subendocardial viability ratio did not relate to SCORE. Endothelial function indices showed no consistent relation to cardiac target organ damage. The agreement between the different methods for evaluating indices of macrovascular and microvascular endothelial function was weak. In conclusion, indices of macrovascular and microvascular endothelial function relate to cardiovascular mortality risk. Their use may improve cardiovascular risk prediction in hypertension. However, methods representing different vascular beds show little interrelationship and are not interchangeable, which may depend on different pathogenetic mechanisms representing different aspects of future cardiovascular risk.

*Trial registry*: NCT02901977

## Introduction

Impaired endothelial function can detect early disturbances in vascular function that precede symptomatic atherosclerotic disease. Changes in forearm blood flow during intra-arterial infusion of acetylcholine (Ach) and sodium nitroprusside (SNP) to induce endothelium dependent and endothelium independent vasodilatation, respectively, have been used to assess endothelial function representing mainly resistance arteries, and reveal that endothelial dysfunction is associated with risk of future atherosclerotic cardiovascular events in the general population and in hypertension [1, 2]. Endothelial function can also be evaluated by non-invasive techniques. Forearm post-ischemic hyperaemia induced flow mediated vasodilatation (FMD) is often used to assess endothelial function representing conduit arteries. FMD is also related to invasively assessed coronary artery endothelial function and coronary flow reserve [3, 4]. Impaired FMD predicts risk of future cardiovascular events in the general population, and in patients with hypertension or with atherosclerotic disease in some [5–7], but not all [8, 9] studies. Beta 2-adrenoceptor agonist stimulation induces endothelial dependent vasodilation and changes pulse wave reflection, which can be expressed as the change in augmentation index derived from the first reflected wave, or from the relative height of the first diastolic reflective wave [2, 10]. This non-invasive technique assesses endothelial function representing resistance arteries. A blunted response is associated with coronary artery disease, hypercholesterolemia, and diabetes [10, 11].

Non-invasive methods are also available to assess microvascular function in man. Skin microvascular reactivity can be evaluated by laser Doppler fluxmetry (LDF) and transdermal iontophoretic application of Ach and SNP to represent endothelium dependent and endothelium independent vasodilatation, respectively, or by the maximum hyperaemic response to local heating or following arterial occlusion [12, 13]. Hypertensive patients show impaired forearm skin microvascular reactivity with signs of endothelial dysfunction together with structural and functional capillary rarefaction [12, 14]. In addition, disturbed skin microvascular reactivity is associated with coronary heart disease and incident type 2 diabetes [15, 16]. Microvascular function in the coronary circulation can be assessed by the subendocardial viability ratio (SEVR), which can be calculated from pulse wave analysis and tonometry, to reflect an index of myocardial oxygen supply and demand. A lower SEVR, representing impaired subendocardial perfusion, is associated with a reduced coronary flow reserve [17, 18] and can predict cardiovascular mortality and end stage renal disease in patients with type 1 diabetes and in chronic kidney disease [19, 20].

A simple way to assess cardiovascular risk is important to identify people at high risk, and to offer them appropriate prevention strategies. Hypertension-induced target organ damage such as left ventricular (LV) hypertrophy and its geometric pattern is a strong independent risk factor for future cardiovascular morbidity and mortality, and for all cause mortality [21, 22]. Another means of risk prediction is to use risk algorithms, such as the European Systematic coronary risk evaluation (SCORE) algorithm [23] or the US Framingham risk score [24]. SCORE is based on cardiovascular risk factors, stratified for different regions and countries in Europe, and estimates a 10-year risk for a fatal cardiovascular event [23]. Evidence suggests that SCORE provides better cardiovascular risk prediction in European populations than the Framingham risk score [25].

Endothelial structure and function in microvascular and macrovascular beds differ, as well as the response to stimulation due to differences in the endothelial surface [26]. However, the interrelation between non-invasive methods to assess macrovascular and microvascular endothelial function in different vascular beds, and specifically skin microvascular reactivity and coronary microvascular function, has not been well studied in hypertension. Simultaneous assessment by different methods may be particularly important as microvascular dysfunction may be a prognostic biomarker while macrovascular endothelial dysfunction may reflect existing atherosclerosis, thus representing different mechanisms and aspects of future cardiovascular risk [27, 28]. Moreover, simultaneous association of macrovascular and microvascular function in different vascular beds to cardiovascular risk has not been well studied in hypertension. Thus, this study aimed to investigate the interrelationship of four methods to evaluate macrovascular and microvascular endothelial function in different vascular regions. Second, we wanted to relate indices of endothelial and microvascular function to cardiovascular risk, as assessed by SCORE and by signs of hypertensive heart disease.

## Methods

### Study design and subjects

The Doxazosin–ramipril study included women and men 18 years or older with untreated mild-to-moderate primary hypertension (office systolic blood pressure 141–180 mmHg and/or diastolic blood pressure 91–110 mmHg) to evaluate the effects of treatment with ramipril or doxazosin during 12 weeks on vascular function, as described in detail elsewhere [29]. Patients with ischemic heart disease, congestive heart failure, arrhythmias, or diabetes mellitus were excluded. The current study reports on investigations performed with no previous antihypertensive drug treatment for at least 4 weeks (63 were previously never treated for hypertension). Patients arrived for the investigations in the morning after overnight fasting, and were asked to refrain from caffeine containing beverages, fruit juices or vitamin C, and any other medication influencing endothelial function. The studies were performed in the supine position following a 20 min period of rest in a quiet room kept at a constant temperature of 21–24 °C. To avoid pharmacological interference on the vascular function protocols the examinations were performed on two consecutive days.

Body mass index (in kg/m^2^) was calculated as weight/height^2^. Routine biochemistry was analysed by standard procedures in fasting blood samples. Estimated glomerular filtration rate was calculated by the CDK-EPI formula [30]. Low density lipoprotein cholesterol was calculated according to Friedewald’s formula. The urine albumin-to-creatinine ratio was calculated by urine albumin (mg/L)/urine creatinine (mmol/L), and microalbuminuria was defined as > 2.5 mg/mmol for men and > 3.5 mg/mmol for women.

### Assessment of global cardiovascular risk

The SCORE algorithm was used for global cardiovascular risk prediction [23]. SCORE uses information on age, sex, smoking, systolic blood pressure, and total cholesterol to predict the 10-year risk for cardiovascular mortality in patients 40–65 years of age. We used the electronic version for low-risk countries (where Sweden belongs), which also includes high-density lipoprotein cholesterol for improved risk prediction [31]. There were 11 patients below the age of 40 years, and 15 patients above the age of 65 years, whom we considered being 40 and 65 years of age, respectively, for the purpose of this risk calculation, as generally recommended.

### Echocardiography

Echocardiography was performed to detect signs of cardiac hypertension-induced organ damage, as an additional estimate of future cardiovascular risk [21, 22]. Investigations were made in the supine position by standard procedures by use of a Vivid 7 Dimension device and a phased array 3,5 MHz transducer (Doppler frequency 5.0–3.5 MHz) (GE Medical System, Horten, Norway), as we have previously described [32]. Measurements of LV end systolic and end diastolic dimensions, interventricular septum, and posterior wall thickness were made with M-mode recordings. The Penn convention was used for calculation of LV mass [33], which was corrected for body surface area (i.e. LV mass index), and an LV mass index > 95 g/m^2^ for women and > 115 g/m^2^ for men was considered LV hypertrophy [34]. To assess LV geometric pattern, relative wall thickness was calculated as (interventricular septum thickness + posterior wall thickness)/LV end diastolic diameter, and considered increased if  > 0.42. Left arterial volume indexed for body surface area (ml/m^2^) was calculated as a mean of the four- and two-chamber view measurements. Evaluation of diastolic function was made by pulsed Doppler registrations. The mitral peak flow velocities of the early (*E*) and late (*A*) waves were used for the *E*/*A* ratio calculations. Tissue Doppler echocardiography was performed in the apical four-chamber view, and by pulsed wave Doppler the mitral annular systolic (*s*) and early diastolic (*e*′) velocities were registered. Calculation of the *e*′ mean (mean of the *e*′ septal and *e*′ lateral registrations) was used to assess LV diastolic filling pressures, *E*/*e*′.

### Blood pressures measurements and pulse wave analysis

Brachial blood pressure for the vascular function examinations was obtained in the supine position by an oscillometric device (OMRON 705IT, OMRON Healthcare Co, Ltd, Kyoto Japan) on the right arm with an appropriately sized cuff, as a mean of three readings 1 min apart. Pulse pressure was calculated as systolic minus diastolic blood pressure.

PWA was assessed by applanation tonometry (Millar Instruments, Houston, TX, US) and the SpygmoCor device (AtCor Pty Ltd, West Ryde, NSW, Australia), as described previously [29]. From radial applanation tonometry, the central aortic waveform was calculated using a general transfer function by the device software, and central blood pressure was derived. The carotid-femoral pulse wave velocity (PWV) was calculated as the carotid-to-femoral distance divided by the transit time difference of the carotid and femoral pulse wave propagation. Also the carotid-radial PWV was calculated similarly, to obtain the carotid-femoral/carotid-radial PWV ratio. This has been suggested to illustrate the cross-talk between macrocirculation and microcirculation, where a mismatch in aortic–brachial stiffness results in increased forward wave transmission pressure into the microcirculation and end organ damage [35]. Increased central aortic to peripheral brachial stiffness is a prognostic marker in patients with established atheosclerotic disease and high cardiovascular risk [36].

### Methods to evaluate endothelial function

FMD was measured by post-ischemic hyperaemia in the non-dominant arm, as previously described [29]. Resting basal diameter of the brachial artery was measured for 1 min by a Vivid 7 Dimension ultrasound device with a 9 MHz linear transducer (GE Medical System, Horten, Norway). Thereafter, an inflated pneumatic tourniquet placed around the forearm to a pressure of 250 mmHg for 5 min induced occlusion of the brachial artery. After cuff deflation, maximum change in diameter was achieved by repeated measurements (30, 60 and 90 s). The relative change from baseline diameter was taken as a measure of FMD. After 10 min of additional rest, endothelium independent vasodilation was induced by 0.4 mg sublingual glyceryl trinitrate (GTN; Nitrolingual, G Pohl-Boskamp GmbH & Co KG, Hohenlockstedt, Germany). Relative changes in artery diameter were calculated from rest to 4 min following GTN administration. The endothelial functional index was calculated as the ratio of the FMD/GTN ratio and was used as a measure of the endothelial vasodilation capacity (37). The inter-assay coefficient of variation for FMD in our laboratory is 15% (*n* = 20).

Endothelial dependent vasodilation was also evaluated by applanation tonometry and PWA with beta 2-adrenoceptor agonist stimulation (29). PWA was performed before and 15 and 20 min after 0.25 mg sc terbutaline (Bricanyl, AstraZeneca, Mölndal, Sweden) to evaluate the maximum effect of beta 2-adrenoceptor agonist stimulation. Aortic waveforms were generated by the SphygmoCor software from radial artery applanation tonometry. The change in reflection index (RI) was a measure of endothelial nitric oxide availability, and hence endothelium dependant vasodilatation [2].

SEVR, an index of myocardial oxygen supply and demand, is an indirect measure of subendocardial perfusion capacity [17]. Applanation tonometry and PWA (see above) was used to calculate SEVR, expressed as the ratio of diastolic/systolic pressure integral of the derived aortic pulse wave (18). Coronary flow reserve is often used to express coronary microvascular function, and SEVR is closely related to direct invasive measurements of coronary flow reserve in response to intracoronary adenosine in hypertension [18]. Thus, SEVR may provide a useful assessment of the coronary microcirculation [18].

Forearm skin microvascular reactivity (vasodilatation) was assessed by LDF and 60 s transcutaneous iontophoretic administration (Periflux system 5000, PF 5010 LDPM Unit, PF5010 Temp Unit, and 481-1 Single Probe, Perimed, Järfälla, Sweden) of small amounts of Ach (Sigma-Aldrich AB, Stockholm, Sweden) and SNP (Hospira, Inc., Lake Forest, IL, USA) to represent endothelium dependent and independent vasodilatation, respectively, as described previously [29]. Skin microvascular peak flux was recorded continuously up to 16 min after iontophoresis, and is expressed in arbitrary units. To determine maximum skin microvascular hyperaemia, peak flux was evaluated after local heating of forearm skin to 44°C for 6 min [29].

### Statistics

Data are presented as mean values ± SD or median values and interquartile ranges, as appropriate. Associations were assessed by linear regression and Pearson’s correlation coefficients (*r*). Associations to cardiovascular risk by SCORE (always log transformed) was also assessed in a multivariable logistic regression model including PWV and microalbuminuria, which have been suggested to improve cardiovascular risk prediction, as compared to SCORE alone [38, 39]. All statistical tests were 2-sided and carried out to a significance level (*P*) of < 0.05. The statistical program used was JMP version 13.0 (SAS Institute Inc., Cary, NC, USA).

The size of the current study population originates from the co-primary outcomes in the Doxazosin–ramipril study, which were changes in endothelial function assessed by FMD, and in haemostatic function measured by the generation of thrombin–antithrombin complex, as presented elsewhere [29, 40].

## Results

### General

Baseline characteristics of the 71 participants are presented in Table 1. About one-third were women. Most women were postmenopausal and no one used systemic hormone replacement therapy. There were few smokers, and lipid levels and glucose metabolism were within normal limits. Most patients had normal renal function (chronic kidney disease stage 1, with an estimated glomerular filtration rate ≥ 90 ml/min/1.72 m^2^), while 29 were in stage 2 (60–89 ml/min/1.72 m^2^) and 2 were in stage 3 (30–59 ml/min/1.72 m^2^).

**Table 1:** Baseline characteristics

Female/male (*n*)	26/45
Age (years)	54.5 ± 12.6
Smokers (*n*)	4
Body mass index (kg/m^2^)	26.8 ± 4.7
Office systolic BP (mmHg)	154 ± 10
Office diastolic BP (mmHg)	93 ± 9
Heart rate (beats/min)	61 ± 8
Plasma cholesterol (mmol/L)	5.4 ± 1.1
Plasma HDL cholesterol (mmol/L)	1.4 ± 0.4
Plasma LDL cholesterol(mmol/L)	3.4 ± 0.9
Fasting plasma glucose (mmol/L)	5.4 ± 0.6
Estimated GFR (mL/min/1.73m^2^)	90.4 ± 14.5
UACR (mg/mmol)	0.7 [0.4–1.05]

Data are presented as mean values ± SD or as median and interquartile ranges for 71 patients

*BP* blood pressure, *HDL* high density lipoprotein cholesterol, *LDL* low density lipoprotein cholesterol, *GFR* glomerular filtration rate, *UACR* urine albumin-to-creatinine ratio

Values for indices of endothelial function and LV structure and function are presented in Table 2. There were 19 patients (5 women and 14 men) out of the 61 with available echocardiographic data with LV hypertrophy. The distribution of LV geometric pattern is shown in Table 2. No patient had reduced (< 40%) LV ejection fraction.

**Table 2:** Indices of endothelial function in different vascular beds and selected echocardiographic measurements

FMD (%)	5.8 ± 4.3
GTN (%)	14.7 ± 6.9
Endothelial function index	0.48 ± 0.47
RI change (%)	−7.0 ± 3.0
SEVR	169 ± 24
Ach peak flux (PU)	33.3 [18.8–60.9]
SNP peak flux (PU)	55.5 [36.6–82.2]
Ach peak flux/SNP peak flux	0.58 [0.39–0.85]
Maximum hyperaemia (PU)	60.5 [39.9–78.1]
LV mass index (g/m^2^)	103 ± 32; range 56.5–192.1
Relative wall thickness	0.38 ± 0.1; range: 0.22–0.68
LV geometric pattern	
Normal geometry (*n*)	31
Concentric remodeling (*n*)	11
Eccentric hypertrophy (*n*)	19
Concentric hypertrophy (*n*)	0
*E*/*A*	1.1 ± 0.4
*E*/*e*′	8.3 ± 1.9
Left atrial volume (ml/m^2^)	31 ± 9

Data from 61–69 patients presented as mean values ± SD, or median values and interquartile ranges

*FMD* forearm flow mediated vasodilatation, *GTN* forearm glycerine trinitrate induced vasodilatation, *FMD/GTN* endothelial functional index, *RI (%)* relative change in reflection index by beta 2-adrenoceptor agonist stimulation, *SEVR* subendocardial viability ratio, *Ach peak flux* acetylcholine induced skin microvascular reactivity, *SNP peak flux* sodium nitroprusside induced skin microvascular reactivity, *Maximum hyperaemia* heat induced skin microvascular reactivity, *PU* perfusion units, *LV* left ventricular, *RWT* left ventricular relative wall thickness, *E/A* peak velocity flow in early diastole divided by peak velocity flow in late diastole, *E/e*′ peak velocity flow in early diastole (*E*) divided by mitral annular early diastolic velocity (*e*′)

### Relations between methods to evaluate endothelial function

Measurements of endothelial function in various vascular beds and their interrelations are presented in Table 3. The relations between indices of endothelial function in the forearm, the coronary microcirculation assessed by SEVR, and the skin microcirculation were, at most, weak. However, there was a trend for SEVR to relate to endothelial function index, and the RI change tended to relate to GTN induced vasodilatation and to skin microcirculatory responses to Ach peak flux.

**Table 3:** Relations between various indices of endothelial function

	GTN	EFI	RI change	SEVR	Ach max	SNP max	Ach/ SNP	Ach (Δ%)	SNP (Δ%)	Heat max	Heat (Δ%)
FMD	**0.19** 0.13	**0.66** < 0.001	**0.17** 0.18	**0.17** 0.18	** 0.15** 0.25	** 0.04** >0.5	** 0.10** 0.43	**0.06** >0.5	**0.05** >0.5	** 0.14** 0.27	**0.15** 0.26
GTN	–	** 0.43** < 0.001	**0.25** 0.06	**0.06** >0.5	** 0.21** 0.091	** 0.15** 0.26	** 0.11** 0.42	** 0.25** 0.044	** 0.07** >0.5	** 0.13** 0.31	**0.04** >0.5
EFI		–	** 0.04** >0.5	**0.22** 0.090	**0.02** >0.5	**0.00** >0.5	** 0.03** >0.5	**0.19** 0.14	**0.07** >0.5	** 0.07** >0.5	**0.14** 0.29
RI change			–	** 0.02** >0.5	** 0.24** 0.058	** 0.04** >0.5	** 0.22** 0.080	** 0.26** 0.036	** 0.10** 0.46	** 0.16** 0.19	** 0.11** 0.37
SEVR				–	** 0.10** 0.43	** 0.03** >0.5	** 0.09** 0.47	** 0.06** >0.5	**0.08** >0.5	** 0.11** 0.40	** 0.07** >0.5
Ach max					–	**0.39** 0.001	**0.58** < 0.001	**0.52** < 0.001	**0.20** 0.12	**0.61** < 0.001	**0.00** >0.5
SNP max						–	** 0.38** 0.015	**0.20** 0.100	**0.61** < 0.001	**0.86** < 0.001	**0.29** 0.019
Ach/ SNP							–	**0.28** 0.018	** 0.35** 0.004	**0.31** 0.011	** 0.06** >0.5
Ach (Δ%)								–	**0.20** 0.105	**0.34** 0.005	**0.48** < 0.001
SNP (Δ%)									–	**0.21** 0.088	**0.54** < 0.001
Heat max										–	**0.62** < 0.001

Linear regression analyses with correlations coefficients (*r*; in bold) and significance values

*FMD* flow mediated vasodilatation, *GTN* glycerine trinitrate induced vasodilatation, *EFI* endothelial function index (i.e. FMD/GTN), *RI (%)* relative change in reflection index, *SEVR* subendocardial viability ratio, *Ach max and SNP max* maximum acetylcholine and sodium nitroprusside induced microvascular reactivity, *Ach (Δ%) and SNP (Δ%)* peak flux changes after acetylcholine and sodium nitroprusside, *Heat max* maximum heat induced hyperaemia, *Heat (Δ%)* peak flux change after heat-induced hyperaemia

### Endothelial function in different vascular beds in relation to cardiovascular risk

**Fig. 1:**
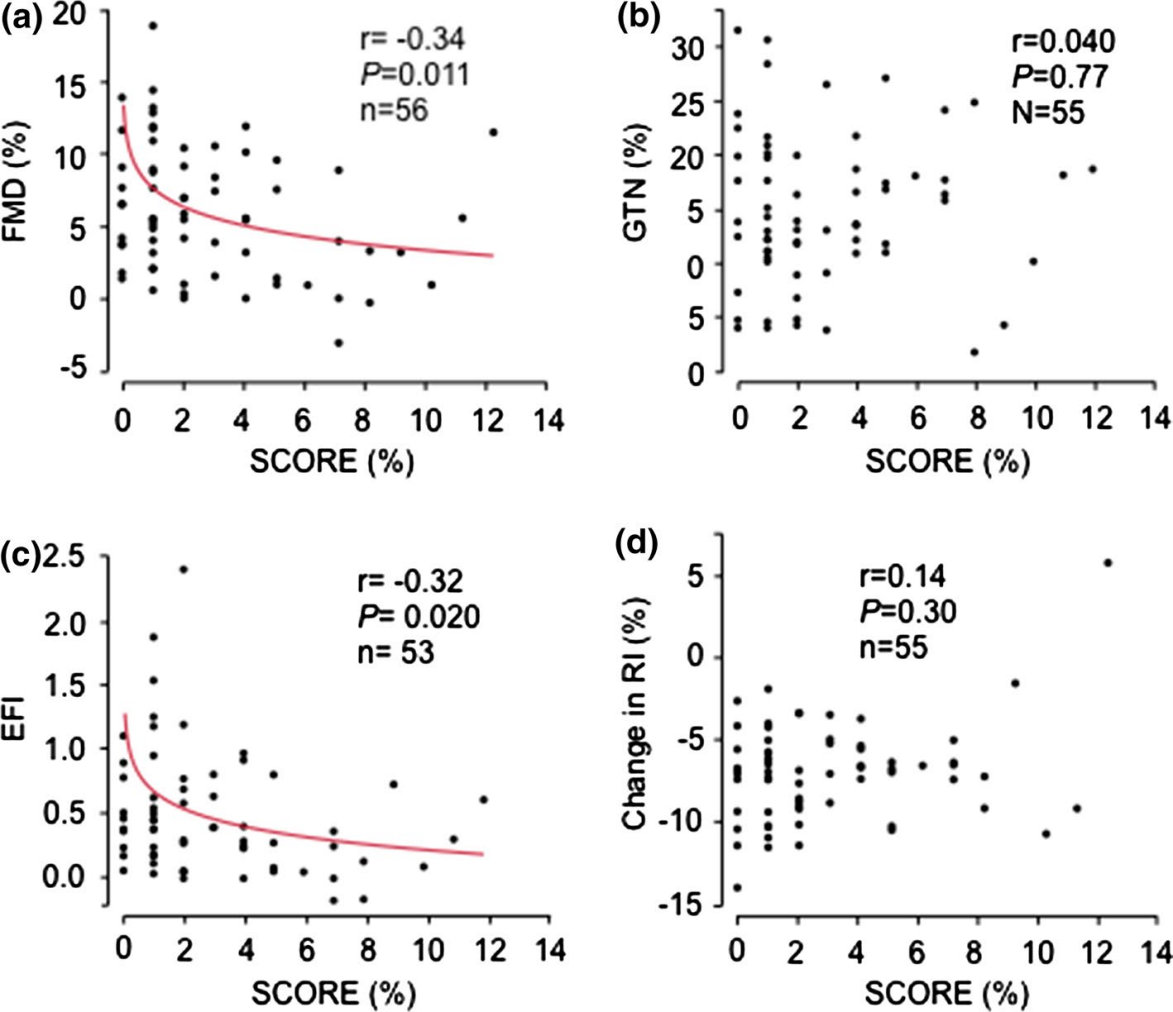
The relations between **a** flow mediated vasodilatation (FMD), **b** glyceryl trinitrate (GTN) mediated vasodilation, **c** endothelial functional index (EFI), and **d** relative change in reflection index (RI) before and after beta 2-adrenoceptor agonist stimulation, and a 10-year-risk for a fatal cardiovascular event, as assessed by the systematic coronary risk evaluation (SCORE)

Endothelium dependent vasodilation (FMD) was inversely related to cardiovascular risk, as assessed by SCORE, while endothelium independent vasodilation (GTN) did not relate to SCORE (Fig. 1a, b). Accordingly, endothelial functional index was inversely related to SCORE (Fig. 1c). There was a trend for a relation between the RI change and SCORE (Fig. 1d).

**Fig. 2:**
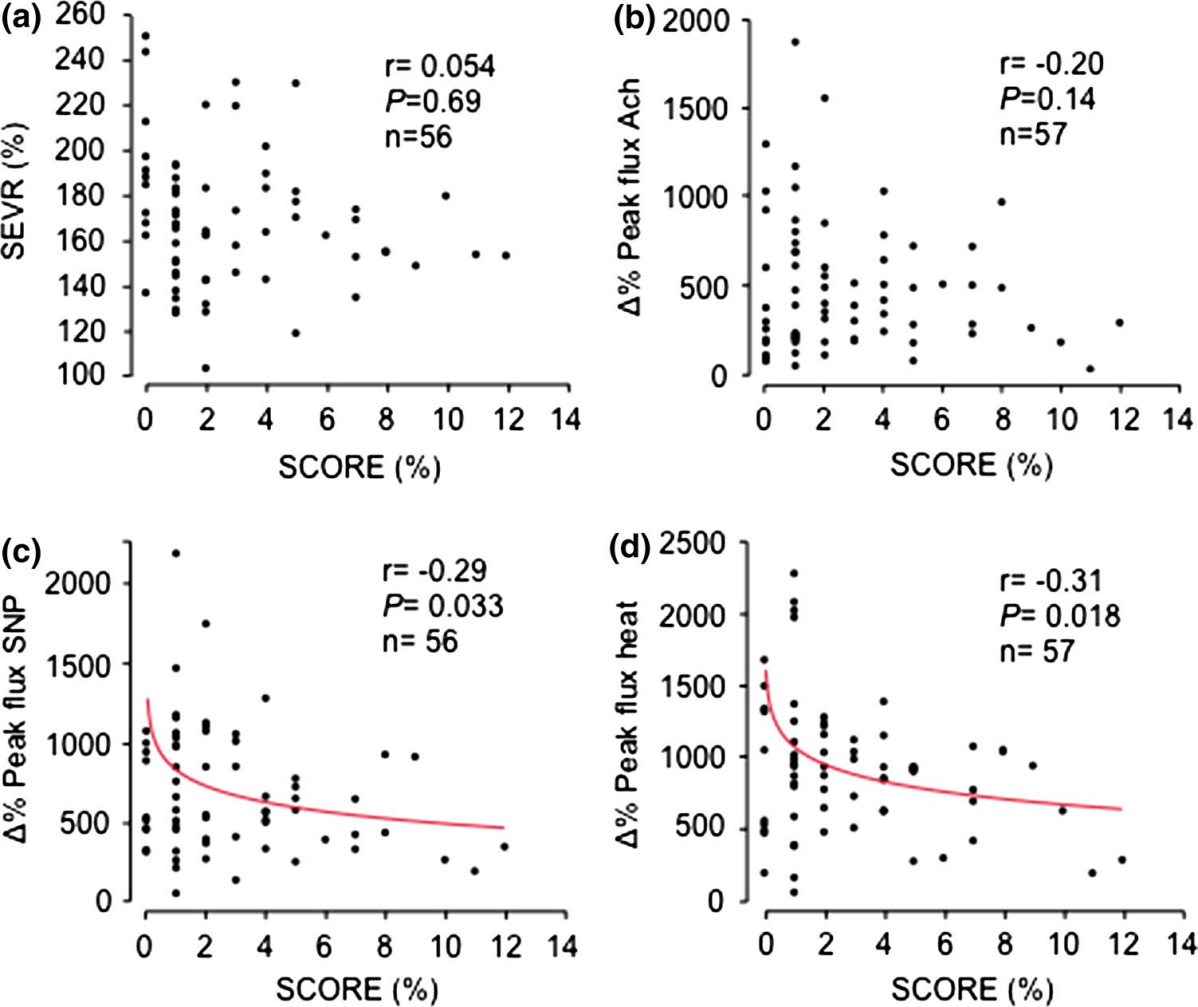
The relations between **a** subendocardial viability ratio (SEVR), **b** relative change in endothelial dependent peak flux (Δ% Peak flux Ach), **c** relative change in endothelial independent peak flux (Δ% Peak flux SNP), and **d** relative change peak flux after maximal hyperaemia (Δ% Peak flux heat), and a 10-year risk for a fatal cardiovascular event, as assessed by the systematic coronary risk evaluation (SCORE)

Coronary microcirculatory function (SEVR) did not relate to SCORE (Fig. 2a). Concerning the skin microcirculation, relative peak flux changes induced by Ach did not relate to SCORE (Fig. 2b). However, relative peak flux changes induced by SNP, and peak flux change after heat induced maximal hyperaemia, all showed inverse relations to SCORE (Fig. 2c, d). Peak LDF (in absolute values) induced by Ach or by SNP were not related to SCORE, and peak flux ratio Ach/SNP did not relate to SCORE (data not shown).

### Endothelial function in relation to signs of hypertensive heart disease

FMD did not relate to LV mass index (data not shown). Accordingly, there were no differences in responses to FMD or GTN when comparing patients without or with LV hypertrophy (6.2 ± 4.5 and 5.4 ± 3.2%, *P* = 0.41 for FMD %, and 15.2 ± 7.7 and 13.5 ± 5.8%, *P* = 0.35, for GTN %, respectively; mean values ± SD). Furthermore, FMD did not relate to relative wall thickness or to indices of diastolic function (i.e. *E*/*A*, *E*/*e*′, or left atrial volume; data not shown). However, endothelial functional index tended to be inversely related to left atrial volume (*r* =  0.23, *P* = 0.087) but not to relative wall thickness, *E*/*A*, or *E*/*e*′ (data not shown). There was a trend for improvement of SEVR to the reduction of *E*/é ratio (*r* =  0.21, *P* = 0.101). However, there were no relations between indices of skin microvascular function (i.e. Ach and SNP peak flux, relative peak flux changes by Ach and SNP, or relative peak flux change after maximal hyperaemia) and LV mass index or with indices of diastolic function (data not shown).

### Endothelial function in relation to indices of arterial stiffness

**Fig. 3:**
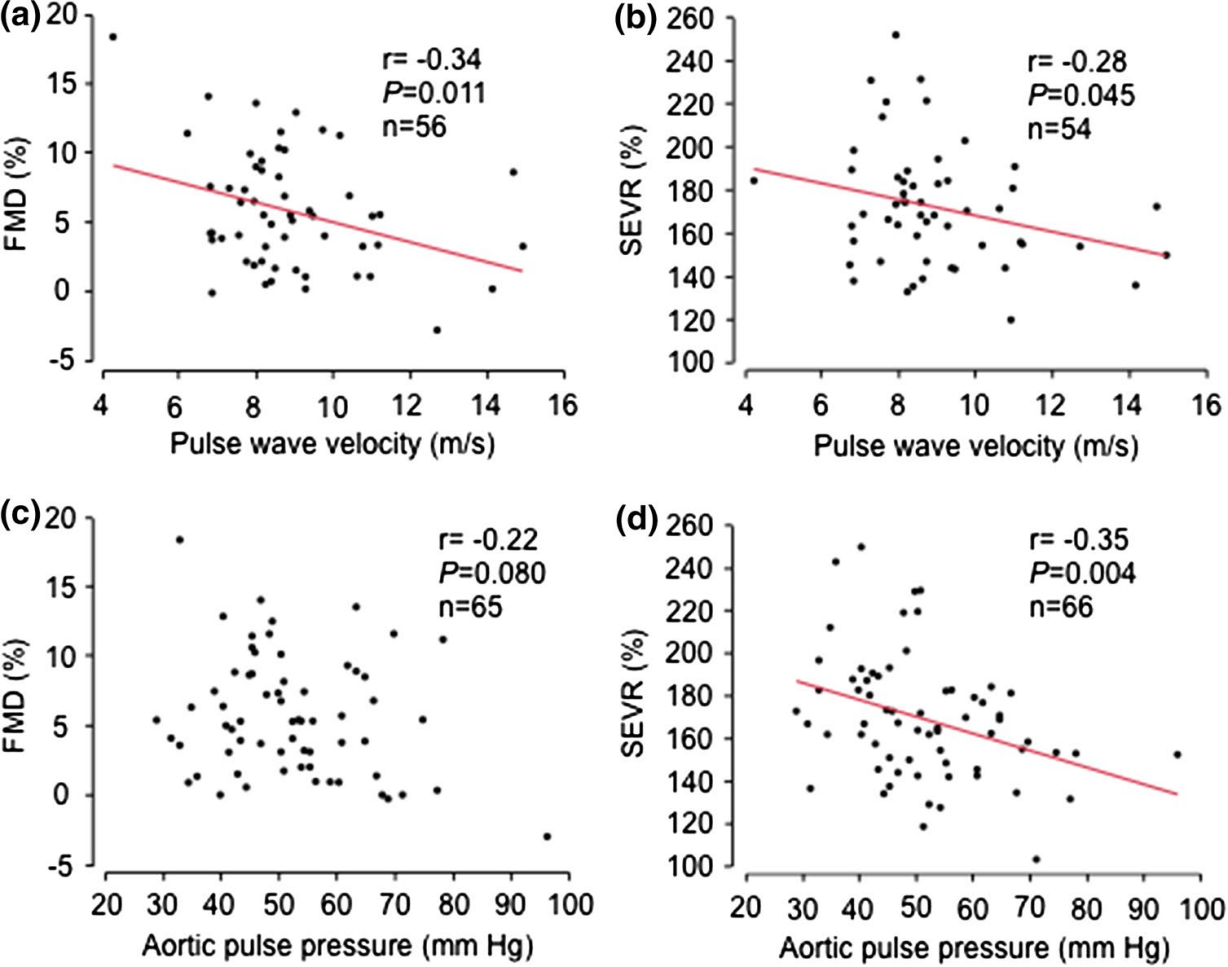
The relations between **a** flow-mediated vasodilatation (FMD), **b** subendocardial viability ratio (SEVR), and carotid-femoral pulse wave velocity, and **c** flow mediated vasodilatation (FMD), and **d** subendocardial viability ratio (SEVR), and aortic pulse pressure

FMD was inversely related to carotid-femoral PWV (Fig. 3a), while GTN induced vasodilatation did not relate to PWV (*r* =  0.11, *P = *0.42). However, endothelial functional index (*r* =  0.05, *P = *0.74) and the RI change (*r* = 0.10, *P = *0.47) failed to relate to PWV. SEVR was inversely related to PWV (Fig. 3b). FMD tended to relate inversely to central aortic pulse pressure (Fig. 3c) but did not relate to brachial pulse pressure (*r* =  0.11, *P* = 0.37). However, endothelial functional index (*r* =  0.14, *P* = 0.27) and the RI change (*r* =  0.12, *P* = 0.33) did not relate to aortic pulse pressure. SEVR was related to aortic pulse pressure (Fig. 3d) and to brachial pulse pressure (*r* =  0.38, *P* = 0.002).

In the skin microcirculation, Ach and SNP peak flux did not significantly relate to PWV (*r* = 0.22, *P* = 0.101, and *r* = 0.06, *P* = 0.66, respectively). However, SNP peak flux related to aortic pulse pressure (*r* = 0.26, *P* = 0.035), and tended to relate to brachial pulse pressure (*r* = 0.23, *P* = 0.061). Ach peak flux tended to relate to aortic pulse pressure (*r* = 0.21, *P* = 0.094) but not to brachial pulse pressure (*r* = 0.18, *P* = 0.13). The relative peak flux changes by Ach and SNP, heat-induced peak flux, and the peak flux change after heat-induced maximal hyperaemia were not related to indices of arterial stiffness (i.e. PWV, aortic pulse pressure, or brachial pulse pressure) (data not shown).

**Fig. 4:**
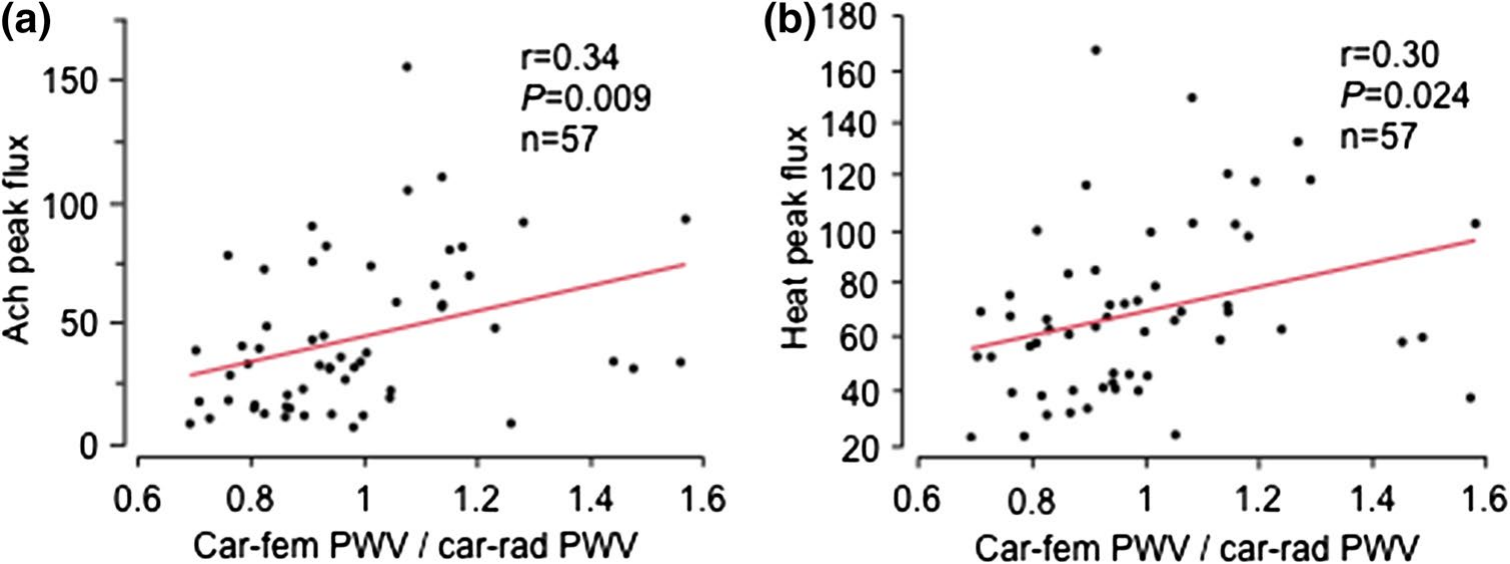
The relations between **a** acetylcholine induced peak flux and **b** peak flux after heat-induced maximum hyperaemia (heat peak flux), and the carotid-femoral to carotid-radial pulse wave velocity ratio (car-fem PWV/car-rad PWV)

Indices of skin microcirculation (Ach peak flux and maximum peak flux after local heating) were related to the carotid-femoral PWV/carotid-radial PWV ratio (Fig. 4a, b), while SNP peak flux did not relate (*r* = 0.06, *P* > 0.5). There were no relations between indices of skin microcirculation and the aortic pulse pressure/brachial pulse pressure ratio (data not shown).

PWV (*P* = 0.014), but not microalbuminuria (*P* > 0.5), was independently related to SCORE. In a multivariable model, PWW and microalbuminuria were added to SCORE in an attempt to provide a better prediction of future cardiovascular risk. This improved the relations between indices of endothelial function and future cardiovascular risk for FMD (*R* =  0.44, *P* = 0.021) and for SEVR (*R* =  0.44, *P* = 0.022), but not for the RI change or for indices for skin microvascular function (data not shown). Adding LV mass index did not improve the multivariable model (data not shown).

## Discussion

### Main findings

This is the first study comparing simultaneous measurements of macrovascular endothelial function by brachial artery vasodilatation induced by post-ischemic flow mediated hyperaemia (by FMD) and pulse wave reflection (by PWA), skin microvascular function (by LDF and iontophoresis), and coronary microvascular function (using SEVR) in hypertensive patients. Of note, SEVR has not previously been well evaluated in relation to peripheral endothelial vascular function. We show that endothelial dysfunction in macrocirculation and microcirculation is both related to cardiovascular mortality risk (by SCORE). However, the applied methods for investigating macrovascular and microvascular function were poorly interrelated, suggesting that endothelial function and microvascular function assessed in different vascular beds represent different aspects of future cardiovascular risk.

### Comparison of endothelial function in different vascular beds

The simultaneous measurements of indices of endothelial function representing conduit arteries (FMD), resistance arteries (PWA), skin microvascular reactivity (LDF and iontophoresis), and coronary microcirculation (SEVR), all previously reported to be associated with cardiovascular risk [6, 9, 15, 18], showed that these markers of macrovascular and microvascular function were poorly interrelated. We found no relation between FMD and skin microvascular function, also when the potential confounding effects of non-specific vasodilation were taken into account. This is in agreement with observations in healthy subjects, and in patients with diabetes or chronic inflammatory disease [41–43]. A relation between conduit artery endothelial function and skin microvascular reactivity has, however, also been reported [44, 45].

The change in pulse wave reflection assessed by the digital volume pulse has been reported to relate to FMD [46]. However, we found no association between change in RI (here assessed by PWA) and FMD, in support of other findings [2]. Thus, our results in hypertensive patients suggest that endothelial function in conduit arteries, as compared to smaller resistance arteries, might not be equivalent. Furthermore, we observed no consistent relations between RI and skin microvascular function, in consort with findings by others [47]. These mixed findings may, at least in part, be related to differences in methodology and study subjects characteristics. More important, however, the lack of relations between macrovascular and microvascular function could be explained by the fact that endothelial dysfunction in conduit vessels and impaired skin microvascular reactivity might develop independently, due to differences in their pathogenesis [48, 49]. Thus, nitric oxide is the major mediator of endothelium dependent vasodilation in conduit arteries, whereas endothelium derived hyperpolarization factor, prostanoids, and other factors are predominant mediators of this in the skin microcirculation [50].

FMD relates to invasive evaluation of coronary artery endothelial function [3] and to coronary flow reserve [4]. However, the present study appears to be the first evaluation of FMD in relation to SEVR. Circumstantial support to our findings that SEVR and FMD did not relate is the observation that SEVR did not relate to dimethylarginines (circulating markers of endothelial dysfunction) [51]. Furthermore, FMD and coronary microvascular reactivity did not relate in healthy subjects or in patients with atherosclerotic coronary artery disease or microvascular angina pectoris [52]. In addition, SEVR was not related to skin microvascular reactivity in the current study. This is in agreement with results suggesting that skin microvascular reactivity and coronary microvascular reactivity are poorly interrelated [53]. Thus, similar to skin microvascular reactivity, coronary microvascular function assessed by SEVR does not seem to relate to macrovascular endothelial function. Accordingly, microvascular dysfunction may be a prognostic biomarker while macrovascular endothelial dysfunction may reflect existing atherosclerosis, representing different mechanisms and aspects of future cardiovascular risk [27, 28].

### Vascular function in relation to cardiovascular mortality risk

FMD was inversely related to SCORE. Our findings were consistent when adjusted for the potential confounding of endothelium independent non-specific vasodilatation assessed by GTN These results support previous observations that reduced endothelium dependent vasodilatation by FMD is inversely related to cardiovascular risk, as reviewed elsewhere [54]. Our findings also confirm an association between FMD and PWV [55]. Furthermore, we confirm that including PWV and microalbuminuria with SCORE in a multivariable linear regression model can improve the prediction of cardiovascular mortality risk [38, 39]. Thus, our results in hypertensive patients extend previous findings [54] to suggest that conduit artery endothelial function is associated to future cardiovascular mortality.

This study also measured endothelial function by PWA and applanation tonometry, where the RI change after beta 2-adrenoceptor agonist stimulation was taken as a measure of global endothelial function, mainly reflecting resistance arteries [2]. The RI change tended to relate to SCORE, in support of our findings on brachial artery endothelial function. Our results are in agreement with those showing that the RI change was related to cardiovascular risk (by Framingham Risk Score), although those authors did not establish a relation to future cardiovascular events [2, 9]. Whereas FMD may be considered to reflect endothelial function in conduit arteries, the RI change is likely to reflect resistance arteries. Conduit arteries may better reflect atherosclerotic vascular changes with a stronger association to cardiovascular risk than resistance arteries reflecting arteriosclerotic vascular disease more closely, and thus being more closely related to blood pressure per se. This could, at least in part, explain the stronger relation observed for FMD than for the RI change to cardiovascular risk in our study.

Coronary microvascular function estimated by SEVR did not relate to SCORE. SEVR is a validated measure of cardiovascular health in subjects with rheumatoid arthritis, a population with a high prevalence of hypertension [56]. Accordingly, SEVR predicts cardiovascular mortality in patients at high risk [19, 20, 57]. However, compared to invasive methods, SEVR is an indirect measure of coronary microcirculation, and might be influenced by other confounding factors. This could have contributed to our findings. In contrast, we showed that total skin microvascular dilatory capacity (assessed as peak flux change to SNP and peak flux change after heat induced maximal hyperaemia) was related to SCORE, in agreement with other results [13, 15]. This suggests that global microvascular vasodilator capacity may be more important than Ach induced skin microvascular reactivity to predict cardiovascular risk. Accordingly, impaired heat-induced maximal hyperaemia in the skin microcirculation has been suggested to discriminate patients with coronary artery disease better than the response to Ach by iontophoresis [58]. These findings indicate that total skin microvascular reactivity is a marker of cardiovascular risk in hypertension.

### Endothelial function in relation to LV mass and function

Impaired macrovascular endothelial function (assessed by FMD, endothelial function index, or RI change) did not relate to LV mass index or indices of LV diastolic dysfunction. Our results are in agreement with some studies [6, 59]; but weak relations between FMD and LV mass index have also been reported [15]. SEVR and LV mass index were not related, and furthermore skin microvascular reactivity was not related to LV mass index or indices of LV diastolic function. Thus, in contrast to our observed associations between vascular reactivity and SCORE, endothelial function measurements did not relate to LV hypertrophy. This suggests that different risk factors are associated to endothelial dysfunction and to hypertensive heart disease. While LV wall tension (i.e. blood pressure) and neurohormonal activation of the renin–angiotensin–aldosterone system and the sympathetic nervous system may be more important for the development of hypertensive heart disease, glucose and lipid metabolism, sex, and smoking may play a greater role for endothelial dysfunction.

### Vascular function in relation to indices of arterial stiffness

Our results show that FMD related to PWV are in agreement with findings that FMD is related to indices of large arterial stiffness [55, 61]. In the skin microcirculation, SNP peak flux related (and Ach peak flux tended to relate) to aortic pulse pressure. Neither Ach peak flux nor SNP associated to PWV but Ach peak flux and maximum peak flux after local heating related to the carotid-femoral PWV/carotid-radial PWV ratio. A mismatch in aortic–brachial stiffness with higher carotid-femoral PWV than carotid-radial PWV has been shown to predict cardiovascular mortality in patients at very high risk (end stage kidney disease) [36]. Our results extend these findings to patients with uncomplicated hypertension (i.e. at a lower risk) to suggest that the carotid-femoral PWV/carotid-radial PWV ratio might be a sensitive measure to detect aortic–brachial stiffness mismatch. An increased PWV mismatch results in an increased forward wave transmission pressure into the microcirculation causing compensating mechanisms like rarefaction and vasodilatation in skin microcirculation where total skin microvascular dilatory capacity is of importance [35].

### Study strengths and limitations

A strength of this study is the evaluation of macrovascular and microvascular function in different vascular beds and by several established non-invasive techniques simultaneously. We compared skin microvascular reactivity and SEVR to large artery endothelial function in hypertensive subjects, which had not been well studied. In addition, SEVR had not been validated to skin microvascular reactivity in hypertensive patients. Second, we studied hypertensive patients with no concomitant confounding cardiovascular disease, ongoing medication, or hormone replacement therapy. However, the study must be interpreted in the context of its limitations. The study population was small and we did not include invasive assessment of endothelial function, which might be considered more reliable. Second, this cross-sectional study evaluated vascular function in relation to predicted future cardiovascular risk and did not prospectively evaluate cardiovascular events. Finally, the results from this small study do not allow us to elucidate in more detail why these methods to assess vascular function were poorly interrelated. However, endothelial function and microvascular function assessed in different vascular beds might develop independently, at least in part due to several differences in their pathogenesis, and thus represent different aspects of future cardiovascular risk [27, 28].

In conclusion, indices of macrovascular and microvascular endothelial function representing different vascular beds were poorly interrelated and are not interchangeable. However, they were all were related to cardiovascular mortality risk, as estimated by SCORE (but not to signs of hypertension-induced cardiac organ damage). These findings suggest that indices of macrovascular and microvascular endothelial function may depend on different mechanisms and thus represent different aspects of future cardiovascular risk. Different techniques to evaluate endothelial function are poorly interrelated. Thus, a global approach to assess vascular endothelial function is required in the risk assessment of hypertensive patients.
